# Microbial and Nutritional Programming—The Importance of the Microbiome and Early Exposure to Potential Food Allergens in the Development of Allergies

**DOI:** 10.3390/nu10101541

**Published:** 2018-10-18

**Authors:** Bożena Cukrowska

**Affiliations:** Immunology Laboratory, Department of Pathology, The Children’s Memorial Health Institute, Aleja Dzieci Polskich 20, 04-730 Warsaw, Poland; b.cukrowska@ipczd.pl; Tel. +48-22-815-10-91

**Keywords:** microbiome, intestinal microbiota, microbial programming, nutritional programming, allergy, prevention

## Abstract

The “microbiota hypothesis” ties the increase in allergy rates observed in highly developed countries over the last decades to disturbances in the gut microbiota. Gut microbiota formation depends on a number of factors and occurs over approximately 1000 days of life, including the prenatal period. During this period the microbiota helps establish the functional immune phenotype, including immune tolerance. The development of immune tolerance depends also on early exposure to potential food allergens, a process referred to as nutritional programming. This article elaborates on the concepts of microbial and nutritional programming and their role in the primary prevention of allergy.

## 1. Introduction

Allergies are one of the key medical problems in highly developed countries, where the proportion of those affected exceeds 30% and continues to grow [1]. The observed increase in allergy rates is associated with the type of lifestyle and involves excessive cleanliness and antibiotic use, small families, increased Cesarean section (CS) rates, altered dietary habits (increased use of processed foods, ready meals), rapid urbanization, and increasingly limited contact with nature [2,3]. These factors immensely affect the composition of the gut microbiota, which is currently believed to be essential for immune system functioning and the development of immune tolerance. The gut microbiota establishes itself over approximately 1000 initial days of life. During this time, the microbiota programs the baby’s immature immune system [4]. Another key factor determining the composition of gut microbiota is nutrition. The baby’s diet affects the composition of microbiota (e.g., in the breastfed infants, predominantly bifidobacteria occur) and is a source of exposure to potential allergens. Studies show that the diet of both the mother (during pregnancy and lactation) and the baby influence the development of allergies later in life [5].

This article presents the role of the gut microbiota and controlled exposure to food allergens in allergy development as well as the possible preventive measures intended to stem the rise in allergy rates.

## 2. Gut Microbiota and Allergic Conditions

The gut microbiota is a complex of microorganisms colonizing the mucous membranes (mainly the intestinal mucosa) and constitutes an integral part of the human body. The number of bacteria colonizing the gastrointestinal tract (10^14^ cells) is comparable to that of all the cells in the body and, as demonstrated by recent calculations, exceeds the number of nucleated human cells 10-fold [6]. However, the microbiome (all the genetic material within the microbiota, i.e., the microbiota genome) is over a hundred-fold bigger than that of the human genome [7]. Modern molecular methods show the intestinal microbiome to be very diverse and individually specific [8,9]. In addition, the composition of the intestinal microbiome undergoes short- and long-term changes throughout life, which are induced by numerous factors such as diet, antibiotic therapy, stress, infections etc. However, the largest modifications of the microbiome, which seem to be permanent, occur during the period of intestinal biocenosis formation [9]. Studies by Lozupone et al. comparing the microbiomes of the citizens of the Republic of Malawi and the United States showed considerable differences in microbiota composition between developing and highly developed countries [10]. By comparing the microbiome of Malavians (Malawi-born individuals who emigrated to the US) and citizens of United States, the study demonstrated that the “Westernized” diet had limited effects on the microbiome established in early childhood. This confirms the theory that the window of opportunity for changing the intestinal microbiome extends over no more than the first two years of a child’s life and any microbiome disturbances (dysbiosis) occurring in this period may result in an abnormal immune system activation and development of pathological conditions such as allergy [3,4].

Studies show that the microbiota in children with allergies are less diversified, with the observed bacterial colonization (predominantly with *Bacteoidetes*, and *Bifidobacterium* and *Lactobacillus* species) reportedly delayed and less numerous in this age group [11,12]. One important fact in microbial programming is that the differences in microbiota composition between children with allergies and healthy children occur during early infancy, even before the first signs of allergy. Assessing 3-week-old neonates who developed allergic symptoms during their first year of life, Kalliomaki et al. demonstrated an increased number of *Clostridium* species, with a concurrent decrease in *Bifidobacterium* genera, compared with healthy children [13]. Moreover, a reduced biodiversity of gut-colonizing bacteria at the age of 1 year was associated with increased allergy rates at age 6 years and the development of asthma at age 7 years [14,15]. Furthermore, a reduced colonization with *Bifidobacterium* and *Lactobacillus* species at the age of 1–2 months induced the development of allergy by the age of 5 years [16].

## 3. The Impact of Gut Microbiota on Immune System Development

Despite developed lymphatic organs, the immune system of neonates is immature. Neonatal lymphocytes are referred to as naïve, i.e., never before exposed to external antigens [17]. Gut-colonizing bacteria are among the first antigens to activate the body’s defense mechanisms, and help seal the intestinal barrier, establish immune tolerance, and modify the body’s response to potential allergens [18]. The gut microbiota forms the first line of defense against pathogens, activates the synthesis of secretory immunoglobulins A (IgA), and increases the expression of the proteins (e.g., zonulin, occludin) that form intraepithelial junctions.

Studies carried out on gnotobiotic experimental models demonstrated that in contrast to conventionally raised mice, germ-free (GF) mice have hypoplastic Peyer’s patches and decreased number of both IgA-secreting plasma cells and lymphocytes located in the lamina propria [19,20]. Colonization of GF animals with components of the gut microbiota induced production of secretory IgA, which are natural antibodies reacting with a wide spectrum of microorganisms and food molecules including allergens. Recently, using transmission electron microscopy we have presented that the gut microbiota improves the immature intestinal epithelium of GF mice [20]. Brush borders of GF-mouse enterocytes were irregularly arranged with decreased numbers of cytoskeletal microfilaments and a lack of elongation into the terminal web. Colonization of GF mice with *Lactobacillus* species obtained from the stool of healthy infants significantly improved this condition. In addition, the adherens junctions of *Lactobacillus* colonized mice were significantly elongated and narrow compared with those in the GF mice and resembled those found in mice colonized with physiological microbiota. This fortification of the intestinal barrier was further evident from the increased levels of the zonulin and occludin proteins in *Lactobacillus*-colonized animals [20].

Gut-colonizing bacteria, which react with Toll-like receptors located on the intestinal epithelium and dendritic cells, stimulate also signaling pathways that activate a number of immune effector cells, such as macrophages, B cells, NK cells, helper T cells (Th1 and Th2), cytotoxic T cells, and regulatory T (Treg) cells [18]. Treg cells regulate the immune response and are characterized by a specific cytokine profile. They produce interleukin (IL)-10 and transforming growth factor (TGF)-beta 1. Treg cells are responsible for maintaining Th1/Th2 cytokine equilibrium and the development of immune tolerance [21]. Our results obtained in gnotobiotic animals showed that stimulation of the immune system by defined components of the intestinal microbiota in early ontogeny is followed by the induction of regulatory mechanisms that maintain the stability of both the local mucosal and systemic immunity [19,20]. This is especially important during the infancy period, as the T cell cytokine profile during early life is pro-allergenic (Th2-like), while Th1 capacity to produce cytokines (IL-12, IFN-gamma) is impaired during this period [17].

## 4. Factors Affecting Gut Microbiota Formation

Despite reports demonstrating the presence of bacteria in the amniotic fluid and placenta, which could indicate the formation of the gut microbiota already in the prenatal period [22,23], in 2017 a study contradicting this theory was published [24]. The assertion that the fetal gastrointestinal tract is sterile and the first gut-colonizing bacteria appear during delivery is believed to be true, and the delivery is the time when the newborn is in direct contact with the microorganisms of the mother or environment [24]. However, the child’s intestinal ecosystem may be affected by the mother’s microbiota as early as during the prenatal period, e.g., via low-molecular-weight metabolites, such as butyric acid. During the prenatal period, butyric acid can induce colonocyte proliferation and growth as well as activate adhesion receptors (on the intestinal epithelium) for the bacteria constituting the newborn’s microbiome [25].

In newborns 98% of intestinal bacteria belong to one of these four phyla: *Firmicutes*, *Bacteroidetes*, *Proteobacteria*, and *Actinobacteria* [9]. From the second week of life, the gastrointestinal tracts of breastfed infants contain predominantly bifidobacteria. As the child develops and solids are introduced into its diet, the microbiota composition becomes more diversified [26]. At the age of approximately 2 years, the gut microbiota stabilizes and the proportion of individual types of bacteria is similar to that observed in adults (i.e., predominantly *Bacteroidetes*). There are a number of factors that shape the infant gut microbiota: the mother’s microbiome, gestational age, mode of delivery, hospital environment, diet (both of the mother and the child), hospitalization period, medication, e.g., antibiotics [26,27,28]. The optimal microbiota composition that may reduce the risk of developing an allergy is observed in infants whose mothers are healthy, did not take antibiotics during pregnancy or lactation, as well as infants who were delivered vaginally, breastfed, living in contact with nature, animals, peers, and siblings, with no antibiotics or excessively sanitized living conditions [27].

The 1000-day pre- and postnatal period of gut ecosystem formation marks the formation of the microbiome—a new organ, which programs mainly the function of the immature immune system but also metabolism and the gut–brain axis [29].

## 5. The Impact of the Mode of Delivery on the Gut Microbiome and Allergy Development

During vaginal delivery, the baby is in contact with the microbiota of the mother’s gastrointestinal tract and birth canal and this microbiota is the main source of microorganisms colonizing the newborn [9]. During the first day of life, neonatal intestines are colonized predominantly by facultative anaerobic bacteria (*Escherichia coli* and enterococci), which proliferate in an oxygen-rich infant gut and prepare the conditions for further colonization with bacteria of the genera: *Bifidibacterium*, *Lactobacillus*, *Bacteroides*, and *Clostridium* [26]. One beneficial effect observed in vaginally delivered newborns is a decrease in the number of *Clostridium* bacteria in favor of *Bifidobacterium* bacteria as early as on day 3 of life [30]. Newborns delivered via a CS are deprived of any contact with the mother’s intestinal microbiota, which results in dysbiosis (an imbalance in the composition of the gut microbiota) observed as early as in the first few hours after birth and over the next days. One-day-old CS-born neonates were shown to be colonized predominantly by the microorganisms colonizing the mother’s skin [31]. These 1-day-old neonates had also a smaller number of *Escherichia coli* and *Bacteroides fragilis*, with higher rates of *Clostridium difficile* isolates and hospital-derived bacteria (including antibiotic-resistant strains) [30]. Despite being breastfed, 3-day-old CS-born neonates exhibited no bifidobacteria [31]. It seems that neonatal period dysbiosis observed after CS delivery may lead to disturbances in microbiotic homeostasis in the subsequent months and years of life. Children delivered via CS demonstrated microbiotic abnormalities such as reduced microbiotic biodiversity, delayed and reduced colonization with *Bacteroidetes* also at the age of 3, 6, 12, and even 24 months [32]. Our studies confirmed an absence of *Bacteroidetes* in the gut of preterm, CS-born neonates one week after birth, with this absence persisting for a period of at least 8 weeks independently on supplementation with *Saccharomyces boulardii* [33].

Disturbances of gut microbiota resulting from CS may affect the development of allergies in the future, which is suggested by epidemiological studies. These studies show that CS deliveries correlate with higher incidence of food allergy and asthma [34,35,36,37]. American cohort studies conducted in 136,098 children indicated that the risk of developing asthma by children aged 4.5–6 years was over seven-fold higher (odds ratio (OR) = 7.77, 95% confidence interval (CI): 6.25–9.65) in the presence of additional factors (besides CS), which negatively affect the gut microbiome. These additional factors included antibiotic therapy during pregnancy and infancy as well as a lack of siblings [38].

## 6. The Effect of Gut Microbiome and Breastfeeding on Allergy Development

Breast milk is the best food for infants, providing the child with all essential nutrients (except vitamins D and K), supporting the function of immature gastrointestinal and immune systems and optimally shaping the development of the gut microbiota [39]. The gut of breastfed infants is colonized predominantly by bifidobacteria [26]. The microbiota of formula-fed children is more diversified and includes *Enterobacteriaceae*, *Enterococcus* species, and *Bacteroides* species [9,26].

The most important component of breast milk (absent from cow’s milk) responsible for the formation of the microbiome are human milk oligosaccharides (HMOs) [40]. There are more than 200 currently known HMOs (resistant to digestive enzymes) which become a selective medium for *Bifidobacteria*, inducing their proliferation in the gut, increasing short-chain fatty acid (e.g., butyric acid) levels, and lowering stool pH. HMOs can also bind to specific intestinal epithelial receptors, which prevents the adhesion of pathogenic bacteria.

Recent studies demonstrated that apart from natural prebiotics (HMOs), breast milk also contains live probiotic bacteria from the genera *Lactobacillus* and *Bifidobacterium* [41,42]. The most commonly isolated *Bifidobacterium* species found in breast milk was *Bifidobacterium breve*, and the most commonly isolated *Lactobacilli* were *Lactobacillus salivarius* and *Lactobacillus fermentum*. Therefore, breast milk is a natural synbiotic containing both probiotics and prebiotics. However, not all mothers’ breastmilk contains probiotic bacteria. The breastmilk of over 50% of women yields no bifidobacterial isolates; moreover, the breastmilk of approximately 30% of women contains no *Lactobacillus* species. The microbiotic profile of breastmilk depends on a number of factors, including the composition of the woman’s gut and skin microbiota, the woman’s health, medication (mainly antibiotics), and the type of delivery [43,44,45]. Antibiotic therapy during pregnancy and lactation dramatically lowers the number of *Lactobacillum* and *Bifidobacterium* genera in breastmilk [43]. The breastmilk of women who delivered via CS contains a lower number of bifidobacteria compared with that of women after a vaginal delivery [44]. The breastmilk of obese mothers was shown to contain a lower number of bifidobacteria and higher number of *Staphylococcus* species as well as to exhibit an altered immunomodulatory capacity, which was associated with lowered levels of such immunostimulating factors as TGF-beta2 and soluble CD14 [45].

Despite the observed differences in the composition of breast milk, studies demonstrate that breast-feeding has a beneficial effect on the occurrence of many diseases associated with the improper functioning of the immune system. Reduced immunity, immaturity of the intestinal barrier and intestinal dysbiosis is primarily associated with premature births. Thus, preterm infants are particularly vulnerable to severe infections such as necrotizing enterocolitis and sepsis. It is now believed that mother’s milk is the optimal diet for such newborns. Human milk has been shown to reduce morbidity and mortality in preterm newborns [46] and protects against necrotizing enterocolitis in early life [47].

In studies on the impact of breast feeding on occurrence of allergies exclusive breastfeeding for 3 months was proven to reduce the risk of atopic dermatitis in genetically predisposed children as well as in those without genetic predisposition [48]. The CHILD (Canadian Healthy Infant Longitudinal Development Birth Cohort) study involving 3296 children showed that, compared with exclusive breastfeeding for the first three months, other diets (such as formula feeding (OR = 2.14; 95% CI 1.37–3.35) or mixed feeding (OR = 1.73; 95% CI 1.17–2.57)) increase the risk of developing asthma at the age of 3 years [49]. Moreover, studies by Chu et al. indicated that breastfeeding for the first 6 months may lower the elevated risk of asthma in children born via CS [50].

## 7. Infant Formulas Supplemented with Prebiotics and Probiotics in Allergy Prevention

Formula-fed or mixed-fed children are more prone to developing allergy. Infant formulas are based on cow’s milk, whose composition is fundamentally different from that of human breast milk. This is why, manufacturers supplement infant formulas with bioactive ingredients present in human breast milk, including substances directly affecting the baby’s microbiome, such as oligosaccharides with prebiotic properties or probiotic bacteria derived from human breast milk (e.g., *Bifidobacterium breve*). There are also synbiotic formulas containing both oligosaccharides and probiotic bacteria.

The most thoroughly studied oligosaccharides are a mixture of short-chain galacto-oligosaccharides (scGOS) and long-chain fructo-oligosaccharides (lcFOS) in a 9:1 ratio, at a dose of 8 g per liter. It was demonstrated that supplementation of infant formulas with a scGOS/lcFOS mixture shifts the microbiotic profile in formula-fed infants towards the profile observed in breastfed infants [51,52]. Consequently, these infants were shown to bear an increased number of bacteria from the genera *Bifidobacterium* and *Lactobacillus*. In addition, formula supplementation with scGOS/lcFOS helped resolve post-antibiotic-therapy dysbiosis, lowered stool pH (the pH reached the values similar to those observed in breastfed infants), and made the short-chain fatty acid profile similar to that present in breastfed infants. The use of a synbiotic formula supplemented with scGOS/lcFOS and the probiotic bacteria *Bifidobacterium breve* M-16V in infants born via CS induced elimination of dysbiosis by increasing the number of bifidobacteria [53]. In older, healthy children aged 1–3 years, a 3-month-long diet of synbiotic formula also resulted in an increase in bacteria of the genus *Bifidobacterium* [54].

Moro et al. are the authors of the first randomized placebo-controlled clinical study on the use of scGOS/lcFOS-supplemented infant formulas conducted in infants at risk of developing allergy [55]. The study group was fed with a partially hydrolyzed whey formula supplemented with scGOS/lcFOS at 8 g/L for 6 months. After this period, the risk of developing atopic dermatitis was halved in the group fed a scGOS/lcFOS-supplemented formula. This beneficial effect persisted for at least 2 years. A 2-year-long prospective study by Arslanoglu et al. demonstrated that feeding infants formulas containing extensively hydrolyzed whey and supplemented with scGOS/lcFOS for 6 months not only lowers the rates of atopic dermatitis, but also significantly reduces the number of children with wheezing and urticaria in comparison with those rates in the group receiving formula without prebiotic supplementation [56]. A 5-year follow-up confirmed persistent, long-term benefits of the evaluated dietary intervention [57].

In 2016, World Allergy Organization (WAO) experts issued their guidelines on the use of prebiotics in allergy prevention [58]. These guidelines suggest that all formula-fed infants should receive formulas supplemented with prebiotics. This WAO guideline was based on an analysis of 18 randomized placebo-controlled trials, which demonstrated that supplementing the diet of healthy formula-fed infants with prebiotics during the first year of life lowers the risk of asthma and recurrent wheezing (relative risk (RR): 0.37; 95% CI 0.17–0.80) as well as the risk of food allergy (RR 0.28; 95% CI 0.08–1.00). The published guidelines emphasized that there is no need to provide prebiotic supplementation either to breastfed infants or to lactating mothers.

One year earlier, by analyzing randomized placebo-controlled studies, WAO experts issued a statement suggesting the potential use of probiotics in primary prevention of allergy in high-risk families (in pregnant women, breastfeeding women, and infants) [59]. A 2013 meta-analysis of 25 randomized placebo-controlled clinical studies involving 4031 children showed probiotics administered both pre- and postnatally to be the most effective [60]. Probiotic supplementation was demonstrated to reduce total IgE levels over a long-term (at least 2-year) follow-up and to lower the risk of allergy, but they had no effect on reducing the incidence of asthma. The WAO guideline does not recommend specific strains for primary prevention of allergy. The authors emphasize that the recommendations are conditional and depend on the results of the further research. Recently, Szajewska and Horvath published a meta-analysis of randomized double blind placebo controlled studies, which presented that pre- and/or postnatal supplementation with *Lactobacillus rhamnosus* GG strain does not affect the occurrence of atopic dermatitis, and this strain should not be recommended in allergy prevention [61]. However, it is necessary to remember that probiotic effects are both strain- and population-specific. Thus, the clinical efficacy may be dependent on the strain, but also on a mode of birth (CS or vaginal delivery) or geographical place of birth (northern or southern countries), and these determinants were not taken into account in the meta-analysis. On the other hand, it should be emphasized that the *Lactobacillus rhamnosus* GG strain does not originate from breast milk, only from the intestine. How much it can affect the specific action of probiotics we do not know, and further research is necessary.

## 8. The Effect of Nutritional Programming on Allergy Development

**Figure 1:**
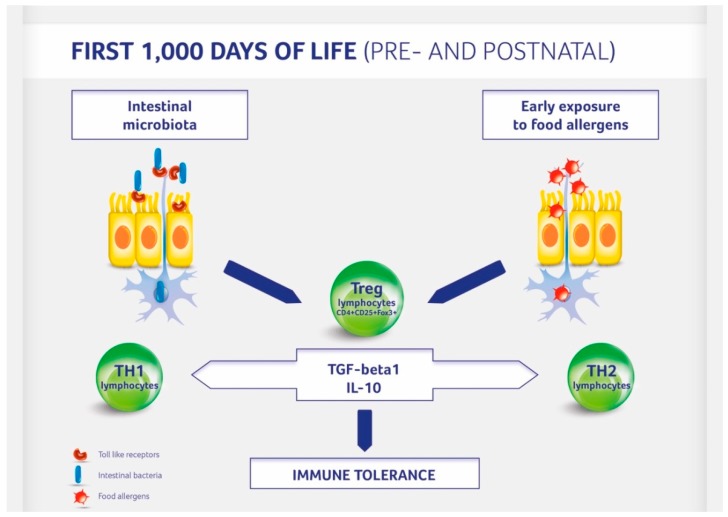
Immune tolerance development in children. Regulatory (Treg) T lymphocytes are activated by the gut microbiota and contact with potential food allergens.

A baby’s diet affects the development of allergies by acting at least bi-directionally (Figure 1). On the one hand food influences the composition of the gut microbiota influencing the development and functioning of the immune system. On the other hand an induction of immune tolerance can be achieved by direct exposure to potential allergens during pregnancy, lactation, and weaning. The contact of the immune system with small doses of allergens activates the formation of Treg lymphocytes [21]. The 20th century was an era of eliminating potential allergens from the diet of both the mother (during pregnancy and lactation) and child (late—often over the age of 1 year—introduction of allergenic foods). The advent of the 21st century completely altered our attitudes toward exposure to potential allergens. The PASTURE study (Protection against Allergy: Study in Rural Environments) of 2014 demonstrated that the less varied the diet during the first year, the higher the risk of developing food allergy at the age of 4, 5, and 6 years [62]. This study also showed a significant reduction of 26% for the development of asthma, with each additional food item introduced in the first year of life. Likewise, a study called LEAP (Learning Early about Peanut Allergy) demonstrated that introducing a potential allergen (in this case: peanuts) at small doses into the diet early (at the age of 4–6 months) reduced the incidence of allergy to this allergen by 80% [63]. Recent studies confirmed that allergen introduction reduced peanut allergy incidence most effectively when peanuts were introduced into the mother’s diet during pregnancy and lactation and then into the infant’s diet [64]. Thus, dietary interventions during gestation and lactation period include a balanced diet without elimination of potential allergens (Table 1). In the postnatal period breastfeeding for a minimum of 4–6 months, the introduction of solid foods according to the recent recommendation of the European Society for Paediatric Gastroenterology Hepatology and Nutrition from week 17 and no later than at week 26 including potential allergens may also have a positive impact on allergy development [65].

The development of cow’s milk (CM) allergy in formula-fed infants may depend on the degree of CM protein hydrolysis [66,67]. Formulas with extensively hydrolyzed proteins and amino acid-based elemental formulas are not intended for healthy infants. Instead, they are for infants with symptomatic allergy to CM proteins. In contrast, partially hydrolyzed formulas (pHF), also called hypoallergenic formulas, which contain partially broken down proteins are especially recommended for children from families at risk of allergy (i.e., when the parents and/or siblings have allergies). It seems that partially hydrolyzed CM proteins can be more effective in induction of specific Treg cells. Gouw et al. identified specific peptides with tolerogenic potential in a whey-based pHF [68]. They presented that partial hydrolysis induced the occurrence of peptides overlapping the specific regions of beta-lactoglobulin, which were found to contain T-cell epitopes with tolerogenic potential. In experimental mice studies partially hydrolyzed whey proteins increased the percentage of Treg cells in the mesenteric lymph nodes leading to a significantly reduced acute allergic skin response to whey [69]. Also, in a clinical trial performed by Boyle et al. the effect of whey-based pHF on Treg cells was investigated. In this randomized, placebo-controlled study after 6 months of intervention, increased percentages of CD4^+^CD25^high^Foxp3^high^ Treg lymphocytes were detected in infants who received pHF compared to the ones who received a standard infant formula. In spite of positive effects on T reg cells, pHF did not prevent eczema in the first year in high-risk infants [70]. In contrast, an update meta-analysis performed by Szajewska and Horvath in 2017 showed that 100% whey-based pHF (manufactured by a single manufacturer), given to infants at risk of allergy development decreased the occurrence of eczema, and reduced the risk of eczema and all allergic diseases among children at high risk of allergy at different time points [66]. Although the certainty of the evidence is low, as the authors underline, such intervention could have beneficial effects in primary prevention of allergies. In a review article by Vandeplas, it is suggested that pHF may be used for all infants, irrespective of family history of allergy [67]; however, at this time, there are no studies confirming the effectiveness of such action.

## 9. Conclusions and Recommendations

Current preventive measures intended to stem the development of allergies focus on inducing immune tolerance via microbial and nutritional programing. Primary prevention of allergies is focused on dietary intervention during both pre- and postnatal periods, and includes a balanced diet without elimination of potential allergens in pregnant and lactating mothers as well as promoting breast feeding for a minimum of 4–6 months, with early introduction of solid foods in the infant diet. To prevent dysbiosis in infants, CS should be only performed when medically indicated, and antibiotic therapy should be limited in both pregnant/lactating women and in infants. In formula- or non-exclusively fed infants, the introduction of synbiotic formulas or those supplemented with prebiotic oligosaccharides can have positive effects on allergy development; however, additional research in this field would be still needed.

## Figures and Tables

**Table 1:** Dietary intervention in pre- and postnatal period which could play role in induction of immune tolerance.

Prenatal Period	Postnatal Period
Balanced and varied diet No elimination of potential allergens The use of probiotics may be considered in families at risk of allergy	Breastfeeding for a minimum 4–6 months Introduction of solid foods starting from week 17; no later than at week 26 No elimination of potential allergens Formulas supplemented with prebiotic oligosaccharides may be considered in formula-fed and non-exclusively fed infants Partially hydrolyzed formulas, optionally supplemented with prebiotics and/or probiotics may be considered in formula-fed and non-exclusively fed infants in families at risk of allergy

## Abbreviations

CI	Confidence interval
CM	Cow milk
CS	Cesarean section
GF	Germ-free
HMOs	Human milk oligosaccharides
IgA (E)	immunoglobulins A (E)
IL	interleukin
lcFOS	long chain fructo-oligosaccharides
OR	Odds ratio
pHF	partially hydrolyzed formula
RR	Relative risk
scGOS	short chain galacto-oligosaccharides
TGF	transforming growth factor
Th	T helper
Treg	regulatory T cells
WAO	World Allergy Organization

## References

[1] Von Mutius E. (1998). The Rising Trends in Asthma and Allergic Diseases. Clin. Exp. Allergy.

[2] Holt P.G., Inouye M., Logan A.C., Prescott S.L., Sly P.D. (2017). An Exposome Perspective: Early-Life Events and Immune Development in a Changing World. J. Allergy Clin. Immunol..

[3] Shreiner A., Huffnagle G.B., Noverr M.C. (2008). The “Microflora Hypothesis” of Allergic Diseases. Adv. Exp. Med. Biol..

[4] Wopereis H., Oozeer R., Knipping K., Belzer C., Knol J. (2014). The First Thousand Days—Intestinal Microbiology of Early Life: Establishing a Symbiosis. Pediatr. Allergy Immunol..

[5] Greer F.R., Sicherer S.H., Burks A.W. (2008). Effects of Early Nutrition Interventions on the Development of Atopic Disease in Infants and Children: The Role of Maternal Dietary Restriction, Breastfeeding, Timing of Introduction of Complementary Foods, and Hydrolyzed Formulas. Pediatrics.

[6] Sender R., Fuchs S., Milo R. (2016). Revised Estimates for the Number of Human and Bacteria Cells in the Body. PLoS Biol..

[7] (2012). Human Microbiome Project Consortium, Structure, Function and Diversity of the Healthy Human Microbiome. Nature.

[8] Eckburg P.B., Bik E.M., Bernstein C.N., Purdom E., Dethlefsen L., Sargent M., Gill S.R., Relman D.A. (2005). Diversity of the Human Intestinal Microbial Flora. Science.

[9] Collado M.C., Cernada M., Baüerl C., Vento M., Pérez-Martínez G. (2012). Microbial Ecology and Host-Microbiota Interactions During Early Life Stages. Gut Microbes.

[10] Lozupone C.A., Stombaugh J.I., Gordon J.I., Jansson J.K., Knight R. (2012). Diversity, Stability and Resilience of the Human Gut Microbiota. Nature.

[11] Björksten B., Naaber P., Sepp E., Mikelsaar M. (1999). The Intestinal Microflora in Allergic Estonian and Swedish 2-year-old Children. Clin. Exp. Allergy.

[12] Abrahamsson T.R., Jakobsson H.E., Andersson A.F., Björkstén B., Engstrand L., Jenmalm M.C. (2012). Low Diversity of the Gut Microbiota in Infants with Atopic Eczema. J. Allergy Clin. Immunol..

[13] Kalliomäki M., Kirjavainen P., Kero P., Salminen S., Isolauri E. (2001). Distinct Patterns of Neonatal Gut Microflora in Infants in Whom Atopy Was Not Developing. J. Allergy Clin. Immunol..

[14] Bisgaard H., Li N., Bonnelykke K., Chawes B.L., Skov T., Paudan-Müller G., Stokholm J., Smith B., Krogfelt K.A. (2011). Reduced Diversity of the Intestinal Microbiota During Infancy Is Associated With Increased Risk of Allergic Disease at School Age. J. Allergy Clin. Immunol..

[15] Abrahamsson T.R., Jakobsson H.E., Andersson A.F., Björkstén B., Engstrand L., Jenmalm M.C. (2014). Low Gut Microbiota Diversity in Early Infancy Precedes Asthma at School Age. Clin. Exp. Allergy.

[16] Sjögren Y.M., Jenmalm M.C., Böttcher M.F., Björkstén B., Sverremark-Ekström E. (2009). Altered Early Infant Gut Microbiota in Children Developing Allergy up to 5 Years of Age. Clin. Exp. Allergy.

[17] Brugman S., Perdijk O., van Neerven R.J., Savelkoul H.F. (2015). Mucosal Immune Development in Early Life: Setting the Stage. Arch. Immunol. Ther. Exp..

[18] Tlaskalová-Hogenová H., Stepánková R., Hudcovic T., Tucková L., Cukrowska B., Lodinová-Zádníková R., Kozáková H., Rossmann P., Bártová J., Sokol D. (2004). Commensal Bacteria (Normal Microflora), Mucosal Immunity and Chronic Inflammatory and Autoimmune Diseases. Immunol. Lett..

[19] Cukrowska B., Kozakova H., Rehakova Z., Sinkora J., Tlaskalova-Hogenova H. (2001). Specific Antibody and Immunoglobulin Responses after Intestinal Colonization of Germ-Free Piglets with Non-Pathogenic Escherichia coli O86. Immunobiology.

[20] Kozakova H., Schwarzer M., Tuckova L., Srutkova D., Czarnowska E., Rosiak I., Hudcovic T., Schabussova I., Hermanova P., Zakostelska Z. (2016). Colonization of Germ-Free Mice with a Mixture of Three Lactobacillus Strains Enhances the Integrity of Gut Mucosa and Ameliorates Allergic Sensitization. Cell. Mol. Immunol..

[21] Akdis C.A., Akdis M. (2014). Mechanisms of Immune Tolerance to Allergens: Role of IL-10 and Tregs. J. Clin. Investig..

[22] Aagaard K., Ma J., Antony K.M., Ganu R., Petrosino J., Versalovic J. (2014). The Placenta Harbors a Unique Microbiome. Sci. Transl. Med..

[23] Collado M.C., Rautava S., Aakko J., Isolauri E., Salminen S. (2016). Human Gut Colonisation May be Initiated in Utero by Distinct Microbial Communities in the Placenta and Amniotic Fluid. Sci. Rep..

[24] Perez-Muñoz M.E., Arrieta M.C., Ramer-Tait A.E., Walter J. (2017). A Critical Assessment of the “Sterile Womb” and “in Utero Colonization” Hypotheses: Implications for Research on the Pioneer Infant Microbiome. Microbiome.

[25] Tan J., McKenzie C., Potamitis M., Thorburn A.N., Mackay C.R., Macia L. (2014). The Role of Short-Chain Fatty Acids in Health and Disease. Adv. Immunol..

[26] Penders J., Thijs C., Vink C., Stelma F.F., Snijders B., Kummeling I., van den Brandt P.A., Stobberingh E.E. (2006). Factors Influencing the Composition of the Intestinal Microbiota in Early Infancy. Pediatrics.

[27] Prescott S.L., Larcombe D.L., Logan A.C., West C., Burks W., Caraballo L., Levin M., Etten E.V., Horwitz P., Kozyrskyj A. (2017). The Skin Microbiome: Impact of Modern Environments on Skin Ecology, barrier Integrity, and Systemic Immune Programming. World Allergy Organ. J..

[28] Martin R., Makino H., Cetinyurek Yavuz A., Ben-Amor K., Roelofs M., Ishikawa E., Kubota H., Swinkels S., Sakai T., Oishi K. (2016). Early-Life Events, Including Mode of Delivery and Type of feeding, Siblings and Gender, Shape the Developing Gut Microbiota. PLoS ONE.

[29] Holzer P., Farzi A. (2014). Neuropeptides and the Microbiota-Gut-Brain Axis. Adv. Exp. Med. Biol..

[30] Dominguez-Bello M.G., Costello E.K., Contreras M., Magris M., Hidalgo G., Fierer N., Knight R. (2010). Delivery Mode Shapes the Acquisition and Structure of the Initial Microbiota Across Multiple Body Habitats in Newborns. Proc. Natl. Acad. Sci. USA.

[31] Biasucci G., Benenati B., Morelli L., Bessi E., Boehm G. (2008). Cesarean Delivery May Affect the Early Biodiversity of Intestinal Bacteria. J. Nutr..

[32] Jakobsson H.E., Abrahamsson T.R., Jenmalm M.C., Harris K., Quince C., Jernberg C., Björkstén B., Engstrand L., Andersson A.F. (2014). Decreased Gut Microbiota Diversity, Delayed Bacteroidetes Colonisation and Reduced Th1 Responses in Infants Delivered by Caesarean Section. Gut.

[33] Zeber-Lubecka N., Kulecka M., Ambrozkiewicz F., Paziewska A., Lechowicz M., Konopka E., Majewska U., Borszewska-Kornacka M., Mikula M., Cukrowska B. (2016). Effect of Saccharomyces boulardii and Mode of Delivery on the Early Development of the Gut Microbial Community in Preterm Infants. PLoS ONE.

[34] Papathoma E., Triga M., Fouzas S., Dimitriou G. (2016). Cesarean Section Delivery and Development of Food Allergy and Atopic Dermatitis in Early Childhood. Pediatr. Allergy Immunol..

[35] Magnus M.C., Håberg S.E., Stigum H., Nafstad P., London S.J., Vangen S., Nystad W. (2011). Delivery by Cesarean Section and Early Childhood Respiratory Symptoms and Disorders the Norwegian Mother and Child Cohort Study. Am. J. Epidemiol..

[36] Guibas G.V., Moschonis G., Xepapadaki P., Roumpedaki E., Androutsos O., Manios Y., Papadopoulos N.G. (2013). Conception via in vitro Fertilization and Delivery by Caesarean Section Are Associated with Paediatric Asthma Incidence. Clin. Exp. Allergy.

[37] Tollånes M.C., Moster D., Daltveit A.K., Irgens L.M. (2008). Cesarean Section and Risk of Severe Childhood Asthma: A Population-Based Cohort Study. J. Pediatrics.

[38] Wu P., Feldman A.S., Rosas-Salazar C., James K., Escobar G., Gebretsadik T., Li S.X., Carroll K.N., Walsh E., Mitchel E. (2016). Relative Importance and Additive Effects of Maternal and Infant Risk Factors on Childhood Asthma. PLoS ONE.

[39] Musilova S., Rada V., Vlkova E., Bunesova V. (2014). Beneficial Effects of Human Milk Oligosaccharides on Gut Microbiota. Benef. Microbes.

[40] Chen X. (2015). Human Milk Oligosaccharides (HMOS): Structure, Function, and Enzyme-Catalyzed Synthesis. Adv. Carbohydr. Chem. Biochem..

[41] Fernández L., Langa S., Martín V., Jiménez E., Martín R., Rodríguez J.M. (2013). The Microbiota of Human Milk in Healthy Women. Cell. Mol. Biol..

[42] Martín R., Jiménez E., Heilig H., Fernández L., Marín M.L., Zoetendal E.G., Rodríguez J.M. (2009). Isolation of Bifidobacteria from Breast Milk and Assessment of the Bifidobacterial Population by PCR-Denaturing Gradient Gel Electrophoresis and Quantitative Real-Time PCR. Appl. Environ. Microbiol..

[43] Soto A., Martín V., Jiménez E., Mader I., Rodríguez J.M., Fernández L. (2014). Lactobacilli and Bifidobacteria in Human Breast Milk: Influence of Antibiotherapy and Other Host and Clinical factors. J. Pediatr. Gastroenterol. Nutr..

[44] Khodayar-Pardo P., Mira-Pascual L., Collado M.C., Martínez-Costa C. (2014). Impact of Lactation Stage, Gestational age and Mode of Delivery on Breast Milk Microbiota. J. Perinatol..

[45] Collado M.C., Laitinen K., Salminen S., Isolauri E. (2012). Maternal Weight and Excessive Weight Gain During Pregnancy Modify the Immunomodulatory Potential of Breast Milk. Pediatr. Res..

[46] Abrams S.A., Schanler R.J., Lee M.L., Rechtman D.J. (2014). Greater Mortality and Morbidity in Extremely Preterm Infants Fed a Diet Containing Cow Milk Protein Products. Breastfeed. Med..

[47] Quigley M., McGuire W. (2018). Formula versus Donor Breast Milk for Feeding Preterm or Low Birth Weight Infants. Cochrane Database Syst. Rev..

[48] Kramer M.S. (2011). Breastfeeding and Allergy: The Evidence. Ann. Nutr. Metab..

[49] Klopp A., Vehling L., Becker A.B., Subbarao P., Mandhane P.J., Turvey S.E., Lefebvre D.L., Sears M.R., Azad M.B., CHILD Study Investigators (2017). Modes of Infant Feeding and the Risk of Childhood Asthma: A Prospective Birth Cohort Study. J. Pediatr..

[50] Chu S., Chen Q., Chen Y., Bao Y., Wu M., Zhang J. (2017). Cesarean Section Without Medical Indication and Risk of Childhood Asthma, and Attenuation by Breastfeeding. PLoS ONE.

[51] Moro G.E., Mosca F., Miniello V., Fanaro S., Jelinek J., Stahl B., Boehm G. (2003). Effects of a New Mixture of Prebiotics on Faecal Flora and Stools in Term Infants. Acta Paediatr..

[52] Knol J., Scholtens P., Kafka C., Steenbakkers J., Gro S., Helm K., Klarczyk M., Schöpfer H., Böckler H.M., Wells J. (2005). Colon Microflora in Infants Fed Formula with Galacto- and Fructo-Oligosaccharides: More Like Breast-Fed Infants. J. Pediatr. Gastroenterol. Nutr..

[53] Chua M.C., Ben-Amor K., Lay C., Neo A.G.E., Chiang W.C., Rao R., Chew C., Chaithongwongwatthana S., Khemapech N., Knol J. (2017). Effect of Synbiotic on the Gut Microbiota of Cesarean Delivered Infants: A Randomized, Double-Blind, Multicenter Study. J. Pediatr. Gastroenterol. Nutr..

[54] Kosuwon P., Lao-Araya M., Uthaisangsook S., Lay C., Bindels J., Knol J., Chatchatee P. (2018). A Synbiotic Mixture of scGOS/lcFOS and Bifidobacterium breve M-16V Increases Faecal Bifidobacterium in Healthy Young Children. Benef. Microbes.

[55] Moro G., Arslanoglu S., Stahl B., Jelinek J., Wahn U., Boehm G. (2006). A Mixture of Prebiotic Oligosaccharides Reduces the Incidence of Atopic Dermatitis During the First Six Months of Age. Arch. Dis. Child..

[56] Arslanoglu S., Moro G.E., Schmitt J., Tandoi L., Rizzardi S., Boehm G. (2008). Early Dietary Intervention with a Mixture of Prebiotic Oligosaccharides Reduces the Incidence of Allergic Manifestations and Infections During the First Two Years of Life. J. Nutr..

[57] Arslanoglu S., Moro G.E., Boehm G., Wienz F., Stahl B., Bertino E. (2012). Early Neutral Prebiotic Oligosaccharide Supplementation Reduces the Incidence of Some Allergic Manifestations in the First 5 Years of Life. J. Biol. Regul. Homeost. Agents.

[58] Cuello-Garcia C.A., Fiocchi A., Pawankar R., Yepes-Nuñez J.J., Morgano G.P., Zhang Y., Ahn K., Al-Hammadi S., Agarwal A., Gandhi S. (2016). World Allergy Organization-McMaster University Guidelines for Allergic Disease Prevention (GLAD-P.): Prebiotics. World Allergy Organ J..

[59] Fiocchi A., Pawankar R., Cuello-Garcia C., Ahn K., Al-Hammadi S., Agarwal A., Beyer K., Burks W., Canonica G.W., Ebisawa M. (2015). World Allergy Organization-McMaster University Guidelines for Allergic Disease Prevention (GLAD-P.): Probiotics. World Allergy Organ J..

[60] Elazab N., Mendy A., Gasana J., Vieira E.R., Quizon A., Forno E. (2013). Probiotic Administration in Early Life, Atopy, and Asthma: A Meta-analysis of Clinical Trials. Pediatrics.

[61] Szajewska H., Horvath A. (2018). Lactobacillus rhamnosus GG in the Primary Prevention of Eczema in Children: A Systematic Review and Meta-Analysis. Nutrients.

[62] Roduit C., Frei R., Depner M., Schaub B., Loss G., Genuneit J., Pfefferle P., Hyvärinen A., Karvonen A.M., Riedler J. (2014). Increased Food Diversity in the First Year of Life is Inversely Associated with Allergic Diseases. J. Allergy Clin. Immunol..

[63] Du Toit G., Roberts G., Sayre P.H., Bahnson H.T., Radulovic S., Santos A.F., Brough H.A., Phippard D., Basting M., Feeney M. (2015). Randomized Trial of Peanut Consumption in Infants at Risk for Peanut Allergy. N. Engl. J. Med..

[64] Pitt T.J., Becker A.B., Chan-Yeung M., Chan E.S., Watson W.T.A., Chooniedass R., Azad M.B. (2018). Reduced Risk of Peanut Sensitization Following Exposure through Breast-Feeding and Early Peanut Introduction. J. Allergy Clin. Immunol..

[65] Fewtrell M., Bronsky J., Campoy C., Domellöf M., Embleton N., Fidler Mis N., Hojsak I., Hulst J.M., Indrio F., Lapillonne A., Molgaard C. (2017). Complementary Feeding: A Position Paper by the European Society for Paediatric Gastroenterology, Hepatology, and Nutrition (ESPGHAN) Committee on Nutrition. J. Pediatr. Gastroenterol. Nutr..

[66] Szajewska H., Horvath A. (2017). A Partially Hydrolyzed 100% Whey Formula and the Risk of Eczema and Any Allergy: An Updated Meta-analysis. World Allergy Organ J..

[67] Vandenplas Y. (2017). Prevention and Management of Cow’s Milk Allergy in Non-Exclusively Breastfed Infants. Nutrients.

[68] Gouw J.W., Jo J., Meulenbroek L.A.P.M., Heijjer S., Kremer E., Sandalova E., Knulst A.C., Jeurink P., Garssen J., Rijnierse A., Knippels L.M.J. (2018). Identification of Peptides with Tolerogenic Potential in a Hydrolyzed Whey-Based Infant Formula. Clin. Exp. Allergy.

[69] Van Esch B.C., Schouten B., de Kivit S., Hofman G.A., Knippels L.M., Willemsen L.E., Garssen J. (2011). Oral Tolerance Induction by Partially Hydrolyzed Whey Protein in Mice is Associated with Enhanced Numbers of Foxp3+ Regulatory T-Cells in the Mesenteric Lymph Nodes. Pediatr. Allergy Immunol..

[70] Boyle R.J., Tang M.L., Chiang W.C., Chua M.C., Ismail I., Nauta A., Hourihane J.O.B., Smith P., Gold M., Ziegler J. (2016). Prebiotic-Supplemented Partially Hydrolysed Cow’s Milk Formula for the Prevention of Eczema in High-Risk Infants: A Randomized Controlled Trial. Allergy.

